# Kinetics of SARS-CoV-2 Specific and Neutralizing Antibodies over Seven Months after Symptom Onset in COVID-19 Patients

**DOI:** 10.1128/Spectrum.00590-21

**Published:** 2021-09-22

**Authors:** Liguo Zhu, Xin Xu, Baoli Zhu, Xiling Guo, Ke Xu, Ci Song, Jianguang Fu, Huiyan Yu, Xiaoxiao Kong, Jiefu Peng, Haodi Huang, Xin Zou, Yuqing Ding, Changjun Bao, Fengcai Zhu, Zhibin Hu, Ming Wu, Hongbin Shen

**Affiliations:** a Jiangsu Provincial Center for Disease Control and Prevention, Nanjing, China; b Department of Epidemiology, Center for Global Health, School of Public Health, Nanjing Medical University, Nanjing, China; c Jiangsu Key Lab of Cancer Biomarkers, Prevention and Treatment, Jiangsu Collaborative Innovation Center for Cancer Medicine, Nanjing Medical University, Nanjing, China; d NHC Key Laboratory of Enteric Pathogenic Microbiology, Jiangsu Provincial Center for Disease Control and Prevention, Nanjing, China; e State Key Laboratory of Reproductive Medicine, Nanjing Medical University, Nanjing, China; f Key Laboratory of Infectious Diseases, School of Public Health, Nanjing Medical University, Nanjing, China; University of Mississippi Medical Center

**Keywords:** antibodies, immune response, natural infection, SARS-CoV-2, virus dynamics

## Abstract

To assess the persistence of severe acute respiratory syndrome coronavirus 2 (SARS-CoV-2) antibodies produced by natural infection and describe the serological characteristics over 7 months after symptom onset among coronavirus disease 2019 (COVID-19) patients by age and severity group, we followed up COVID-19 convalescent patients confirmed from 1 January to 20 March 2020 in Jiangsu, China and collected serum samples for testing IgM/IgG and neutralizing antibodies against SARS-CoV-2 between 26 August and 28 October 2020. In total, 284 recovered participants with COVID-19 were enrolled in our study. Patients had a mean age of 46.72 years (standard deviation [SD], 17.09), and 138 (48.59%) were male. The median follow-up time after symptom onset was 225.5 (interquartile range [IQR], 219 to 232) days. During the follow-up period (162 to 282 days after symptom onset), the seropositive rate of IgM fluctuated around 25.70% (95% confidence interval [CI], 20.72% to 31.20%) and that of IgG fluctuated around 79.93% (95% CI, 74.79% to 84.43%). Of the 284 patients, 64 participants were tested when discharged from hospital. Compared with that at the acute phase, the IgM/IgG antibody levels and IgM seropositivity have decreased; however, the seropositivity of IgG was not significantly lower at this follow-up (78.13% versus 82.81%). Fifty percent inhibitory dilution (ID_50_) titers of neutralizing antibody for samples when discharged from hospital (geometric mean titer [GMT], 82; 95% CI, 56 to 121) were significantly higher than those at 6 to 7 months after discharge (GMT, 47; 95% CI, 35 to 63) (*P *< 0.001). After 7 months from symptom onset, the convalescent COVID-19 patients continued to have high IgG seropositive; however, many plasma samples decreased neutralizing activity.

**IMPORTANCE** The long-term characteristics of anti-SARS-CoV-2 antibodies among COVID-19 patients remain largely unclear. Tracking the longevity of these antibodies can provide a forward-looking reference for monitoring COVID-19. We conducted a comprehensive assessment combining the kinetics of specific and neutralizing antibodies over 7 months with age and disease severity and revealed influencing factors of the protection period of convalescent patients. By observing the long-term antibody levels against SARS-CoV-2 and comparing antibody levels at two time points after symptom onset, we found that the convalescent COVID-19 patients continued to have a high IgG seropositive rate; however, their plasma samples decreased neutralizing activity. These findings provide evidence supporting that the neutralizing activity of SARS-CoV-2-infected persons should be monitored and the administration of vaccine may be needed.

## INTRODUCTION

Coronavirus disease 2019 (COVID-19), caused by the novel severe acute respiratory syndrome coronavirus 2 (SARS-CoV-2), was first reported in Wuhan, China, in December 2019 and has spread around the world violently with ongoing and prolonged high rates of new infections (1). Bearing the continuously heavy burden of life lost and health resource strain, countries have turned to safe and effective vaccines for further and greater release beyond the nonpharmaceutical interventions like physical-distancing that have been taking effect (2, 3). So far, the lessons about immune response to SARS-CoV-2 after natural infection proved the feasibility of achieving herd immunity by vaccine (4, 5), and the long-term immune characteristics of natural infection play a key and referenced role in estimating antibody effects after vaccination, which need more study to support.

In the process of natural immune responses to COVID-19, IgM, expressing first and representing approximately 10% of serum antibodies, shows a great capacity for reacting against the target antigen, while IgG, appearing later, has a high capacity for neutralizing pathogens and existing in human bodies for months (6). In addition, neutralizing antibodies play a vital role in prophylaxis and vaccine development of COVID-19 (7). The long-term characteristics of anti-SARS-CoV-2 antibodies, which include the persistence of antibodies and duration of immune protection affecting the occurrence of reinfection, remain largely unclear. There were some researchers studying the 6-month consequences of antibodies, but the length of observation time and the number of biological samples for testing were not enough (8).

In this study, between 26 August and 28 October 2020, we followed up with 284 convalescent patients with COVID-19 who were infected from 1 January to 20 March 2020 for antibody immunoassay targeting the nucleoprotein and the spike protein and authentic SARS-CoV-2 microplate neutralization assay, the results of which were compared to their antibody levels at the acute phase. The main aims were to check the duration of lasting immune antibodies produced by natural infection and to describe antibody characteristics of convalescent patients grouped by disease severity and age over a longer period.

## RESULTS

### Demographic and clinical characteristics.

The demographic and clinical characteristics of the 284 recovered individuals with laboratory-confirmed COVID-19 are shown in Table 1. Most participants were adults (>18 years old, 96.48%), with a mean age of 46.72 years (standard deviation [SD], 17.09) and more were female (51.41% versus 48.59% male). When hospitalized, nearly half of the participants (44.37%, 126 of 284) were identified as normal cases, 32.04% (91 of 284) were asymptomatic cases, 20.42% (58 of 284) were mild cases, and the rest (3.17%, 9 of 284) was severe/critical cases. Severe/critical cases with a mean age of 61.72 years (SD, 14.04) had a higher proportion of elderly patients (≥60 years) than the other three groups. Most children and adolescents (<20 years old) were asymptomatic patients (9/11, 81.82%). During the acute phase, all of the patients admitted to the intensive care unit (ICU) or inflicted with acute respiratory distress syndrome (ARDS) belonged to the severe/critical type (*n* = 8, *P *< 0.001 and *n* = 2, *P *= 0.001, respectively).

**TABLE 1:** Demographic and clinical characteristics of patients with COVID-19 having accepted serologic tests

Characteristic	No. (%) of patients					*P* ^*a*^
	All patients	Asymptomatic type	Mild type	Normal type	Severe/critical type	
Total	284 (100.00)	91 (32.04)	58 (20.42)	126 (44.37)	9 (3.17)	
Gender						0.332
Male	138 (48.59)	39 (42.86)	28 (48.28)	68 (53.97)	3 (33.33)	
Female	146 (51.41)	52 (57.14)	30 (51.72)	58 (46.03)	6 (66.67)	
Age (mean ± SD)	46.72 ± 17.09	42.94 ± 18.21	51.99 ± 16.44	45.95 ± 15.70	61.72 ± 14.04	0.001^*b*^
Age (yr)						0.001^*b*^
<10	4 (1.41)	3 (3.30)	0 (0.00)	1 (0.79)	0 (0.00)	
10–20	7 (2.46)	6 (6.59)	0 (0.00)	1 (0.79)	0 (0.00)	
20–60	210 (73.94)	63 (69.23)	40 (68.97)	104 (82.54)	3 (33.33)	
≥60	63 (22.18)	19 (20.88)	18 (31.03)	20 (15.87)	6 (66.67)	
Chest CT performance at follow-up, *n* = 283						
Nodules	74 (26.15)	17 (18.89)	16 (27.59)	40 (31.75)	1 (11.11)	0.136
Cysts	8 (2.83)	1 (1.11)	5 (8.62)	2 (1.59)	0 (0.00)	0.063
Fibrosis	42 (14.84)	10 (11.11)	15 (25.86)	15 (11.90)	2 (22.22)	0.048^*b*^
Calcification	22 (7.77)	4 (4.44)	8 (13.79)	8 (6.35)	2 (22.22)	0.055
Ground-glass opacities	21 (7.42)	5 (5.56)	7 (12.07)	8 (6.35)	1 (11.11)	0.362
Patchy shadows	35 (12.37)	8 (8.89)	9 (15.52)	15 (11.90)	3 (33.33)	0.148
Pleural effusion	1 (0.35)	0 (0.00)	0 (0.00)	1 (0.79)	0 (0.00)	1.000
Pleural thickening	30 (10.60)	9 (10.00)	8 (13.79)	12 (9.52)	1 (11.11)	0.794
Typical chest CT characteristics of COVID-19^*c*^	54 (19.08)	12 (13.33)	15 (25.86)	23 (18.25)	4 (44.44)	0.054
ICU when hospitalized	8 (2.82)	0 (0.00)	0 (0.00)	0 (0.00)	8 (88.89)	<0.001^*b*^
ARDS when hospitalized	2 (0.70)	0 (0.00)	0 (0.00)	0 (0.00)	2 (22.22)	0.001^*b*^
Other complications when hospitalized	5 (1.76)	0 (0.00)	0 (0.00)	5 (3.97)	0 (0.00)	0.144

a Chi-square test or Fisher’s exact test as appropriate.

b *P *< 0.05 represents significant difference.

c Typical chest CT characteristics of COVID-19 included ground-glass opacities and patchy shadows.

After they were recruited, 283 of 284 patients were given a bilateral chest computed tomography (CT) scan. Among nonspecific imaging signatures, the most common chest CT finding was pulmonary nodules (26.15%, 74 of 283) followed by fibrosis (14.84%, 42 of 283) and pleural thickening (10.60%, 30 of 283). Regarding typical chest CT characteristics of COVID-19, there were no significant differences among four type of patients in ground-glass opacities and patchy shadows (*P *= 0.362 and 0.148; combined *P *= 0.054).

### IgM/IgG responses against SARS-CoV-2 during this follow-up period.

**FIG 1:**
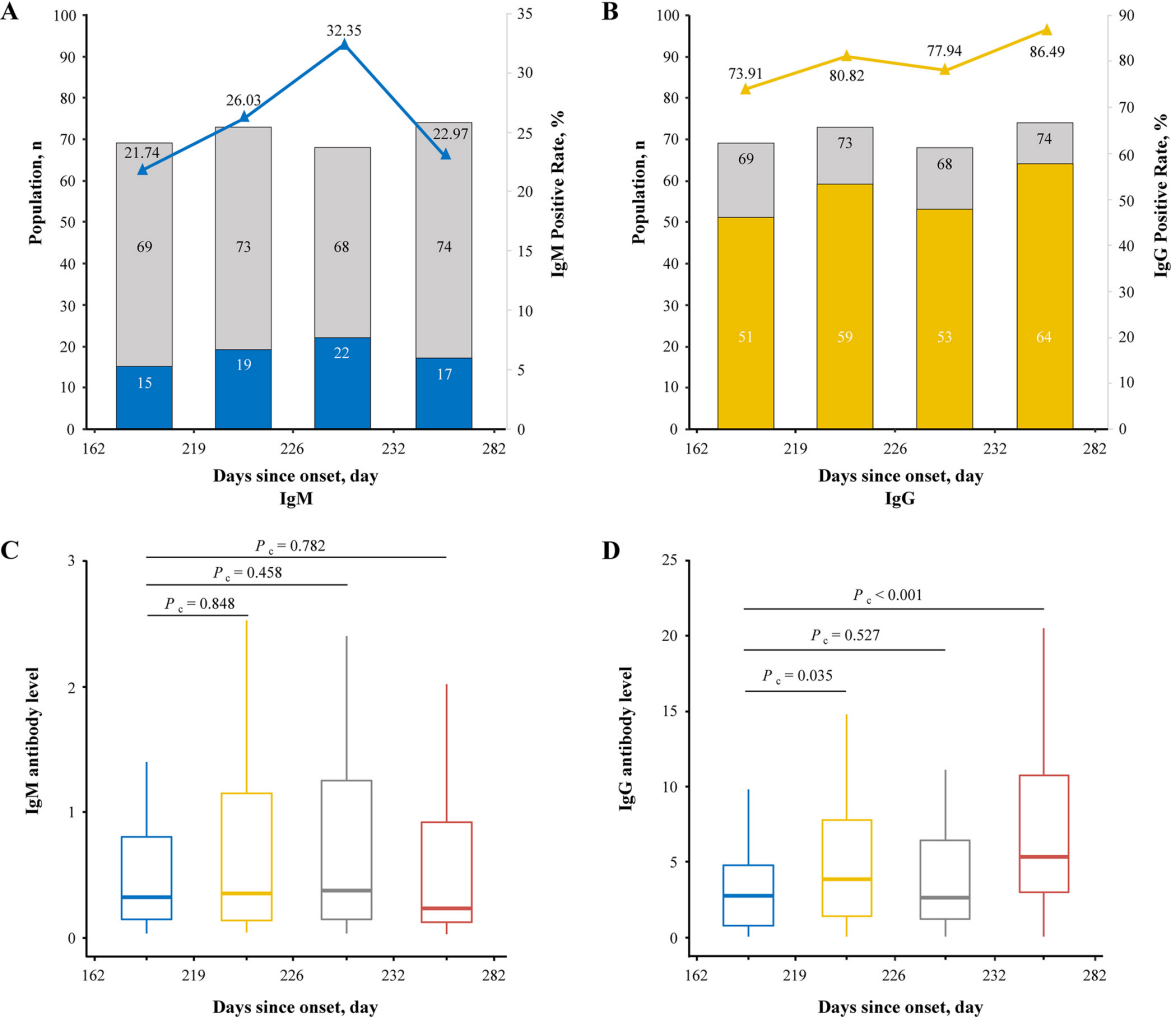
Seropositivity and antibody levels of IgM and IgG against SARS-CoV-2 versus days after symptom onset at follow-up. (A and B) The blue and yellow part of the histogram represents the number of IgM- and IgG-positive participants, respectively, while the gray part represents the total number of individuals in each quartile of days after symptom onset (shown in the left *y* axis); the blue and yellow lines are connected by the same color triangles and denote the positive rate of IgM and IgG in each quartile group, respectively (shown in the right *y* axis). (C and D) Box plots of chemiluminescent immunoassay measurements of IgM and IgG for participants sampled in each quartile of days after symptom onset. *P*_c_ represents that the *P* value was produced by Wilcoxon rank sum (Mann-Whitney) test.

The median time from symptom onset to this follow-up was 225.5 days (interquartile range [IQR], 219 to 232; range, 162 to 282). Stratified by the types of COVID-19, the median time was 223 days (IQR, 212 to 230) for asymptomatic patients, 226.5 days (IQR, 221 to 235) for mild patients, 226 days (IQR, 220 to 231) for normal patients, and 232 days (IQR, 229 to 233) for severe/critical patients, all similar to the median time of all patients. The follow-up period was divided into four intervals according to the days from symptom onset to this follow-up (Fig. 1). During the follow-up period, the seropositive rate of IgG was still much higher than IgM, and interestingly, decay tendency was not observed in both the IgG and IgM seropositive rates, with the former fluctuating around 79.93% (95% confidence interval [CI], 74.79% to 84.43%) and the latter fluctuating around 25.70% (95% CI, 20.72% to 31.20%). Meanwhile, the level of IgM did not show a significant difference between the other three intervals and the interval of 162 to 219 days (*P *= 0.848; *P *= 0.458; *P *= 0.782). Moreover, the levels of IgG in the intervals of 219 to 226 days and 232 to 282 days were significantly higher than that of 162 to 219 days (*P *= 0.035; *P *< 0.001). The population distribution of different disease severity among four interval periods was shown in Fig. S1 in the supplemental material. For the whole group of participants, IgM-seropositive individuals and IgG-seropositive individuals, the normal type was always the largest proportion after 219 days since onset, and the severe/critical type was the minimum during the whole follow-up period.

### IgM/IgG levels over 7 months after symptom onset according to severity of disease.

**FIG 2:**
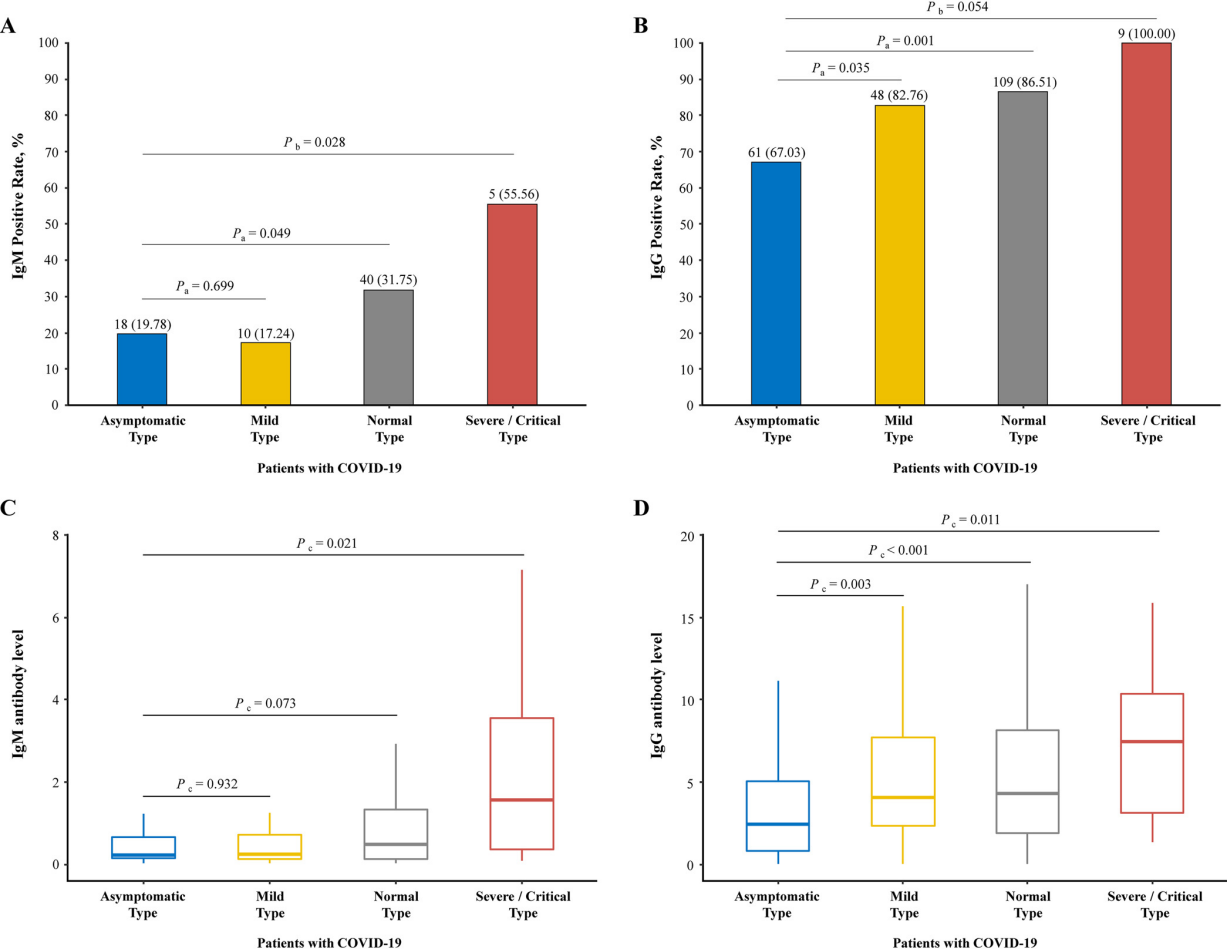
Seropositivity and antibody levels of IgM and IgG against SARS-CoV-2 according to severity of disease at follow-up. (A and B) Bar plots of positive rates of virus-specific IgM and IgG for participants belonging to four type of disease severity in the cohort. (C and D) Box plots of chemiluminescent immunoassay measurements of IgM and IgG for participants belonging to four types. *P*_a_ represents that the *P* value was produced by chi-square test, *P*_b_ represents that the *P* value was produced by Fisher's exact test, and *P*_c_ represents that the *P* value was produced by Wilcoxon rank sum (Mann-Whitney) test.

The level and positivity rate of anti-SARS-CoV-2 IgM/IgG at this follow-up are represented in Fig. 2. The IgG-positive rate was much higher than the IgM rate no matter which type of severity. More than 60% of individuals still remained positive for IgG, with the highest rate of 100% in the severe/critical type followed by normal (86.51%), mild (82.76%), and asymptomatic (67.03%). Compared with the asymptomatic type, the IgG seropositive rates of the mild and normal types were significantly higher (*P *= 0.035 and *P *= 0.001, respectively). Surprising, 55.56% of severe/critical, 31.75% of normal, 17.24% of mild, and 19.78% of asymptomatic patients were still detected for positive IgM at 6 to 9 months after SARS-CoV-2 infection was confirmed. Compared with the asymptomatic type, the IgM seropositive rate of the severe/critical type was significantly higher (*P *= 0.028) while that of the normal type was marginally higher (*P *= 0.049).

Similarly, the level (chemiluminescence values divided by the cutoff [S/CO]) of IgG was much higher than IgM. The level of IgM was 0.23 (IQR, 0.14 to 0.68), 0.25 (IQR, 0.14 to 0.72), 0.49 (IQR, 0.12 to 1.34), and 1.57 (IQR, 0.37 to 3.56) in asymptomatic, mild, normal, and severe/critical patients, respectively. There was statistical significance between the asymptomatic type and severe/critical type (*P *= 0.021); however, the differences between the asymptomatic type and mild and normal types were not statistically significant (*P *= 0.932; *P *= 0.073). Meanwhile, the level of IgG was 2.42 (IQR, 0.81 to 5.06), 4.06 (IQR, 2.33 to 7.87), 4.31 (IQR, 1.89 to 8.19), and 7.46 (IQR, 3.14 to 10.37), respectively, and the differences were statistically significant between the asymptomatic type and the normal and mild and severe/critical types (*P *= 0.003; *P *< 0.001; *P *= 0.011, respectively).

### IgM/IgG levels over 7 months after symptom onset according to age.

**FIG 3:**
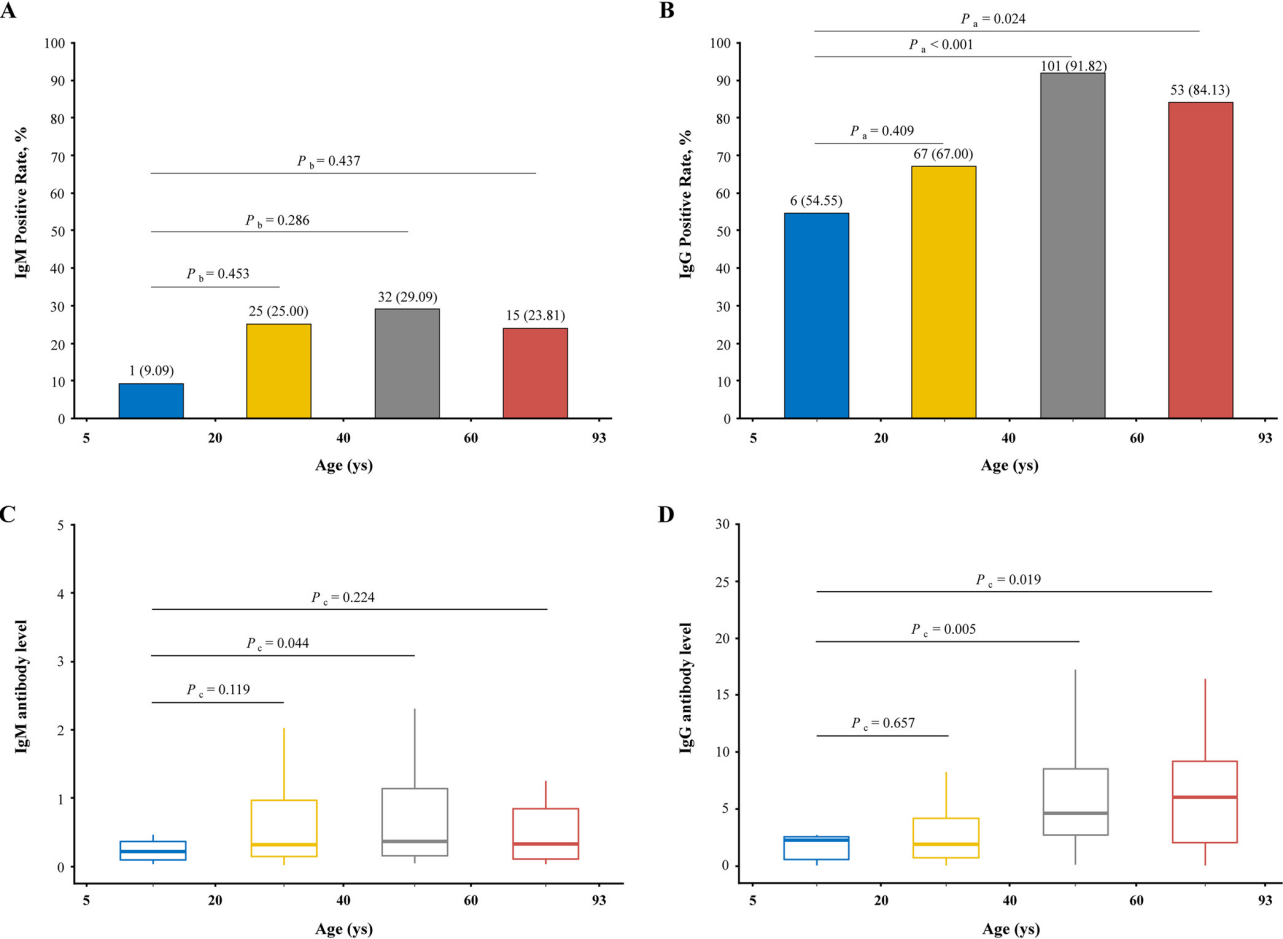
Seropositivity and antibody levels of IgM and IgG against SARS-CoV-2 within age at follow-up. The age range of 284 COVID-19 convalescent participants was 5 to 93 years old and divided into four groups as follows: 5 to 20, 20 to 40, 40 to 60, and 60 to 93 years old. (A and B) Positive rates of IgM and IgG presented with each age interval in bar plots. (C and D) Chemiluminescent immunoassay measurements of IgM and IgG presented with each age interval in box plots. *P*_a_ represents that the *P* value was produced by chi-square test, *P*_b_ represents that the *P* value was produced by Fisher's exact test, and *P*_c_ represents that the *P* value was produced by Wilcoxon rank sum (Mann-Whitney) test.

The age range of participants was 5 to 93 years old and was divided into four intervals as shown in Fig. 3. Both for IgM and IgG, children and adolescents (<20 years old) had the lowest seropositive rates (9.09% and 54.55%) while the participants of 40 to 60 years old had the highest seropositive rates (29.09% and 91.82%). The seropositive rates of the elderly (≥60 years old) were 23.81% for IgM and 84.13% for IgG. Remarkably, the seropositivity of IgM was not significantly different with the reference group of children and adolescents (all *P *> 0.05); however, the IgG seropositive rates of the participants over 40 years old were significantly higher than those of children and adolescents (*P *< 0.001 and *P *= 0.024).

The IgM level of the participants aged 40 to 60 years old was the highest (0.36 [IQR, 0.16 to 1.14]) followed by the elderly (0.33 [IQR, 0.11 to 0.87]), the participants 20 to 40 years of age (0.32 [IQR, 0.14 to 1.04]), and children and adolescents (0.21 [IQR, 0.07 to 0.45]). The IgG level of the elderly was the highest (6.03 [IQR, 1.64 to 9.53]) followed by the participants aged 40 to 60 years (4.59 [IQR, 2.70 to 8.57]), children and adolescents (2.26 [IQR, 0.55 to 2.74]), and the participants aged 20 to 40 years (1.89 [IQR, 0.72 to 4.18]). Compared with children and adolescents, the participants aged 40 to 60 years had a marginally higher (*P *= 0.044) IgM level, and the participants aged 40 to 60 years and the elderly had significantly higher (*P *= 0.005 and *P *= 0.019) IgG levels.

### IgM/IgG change at discharge and follow-up.

**FIG 4:**
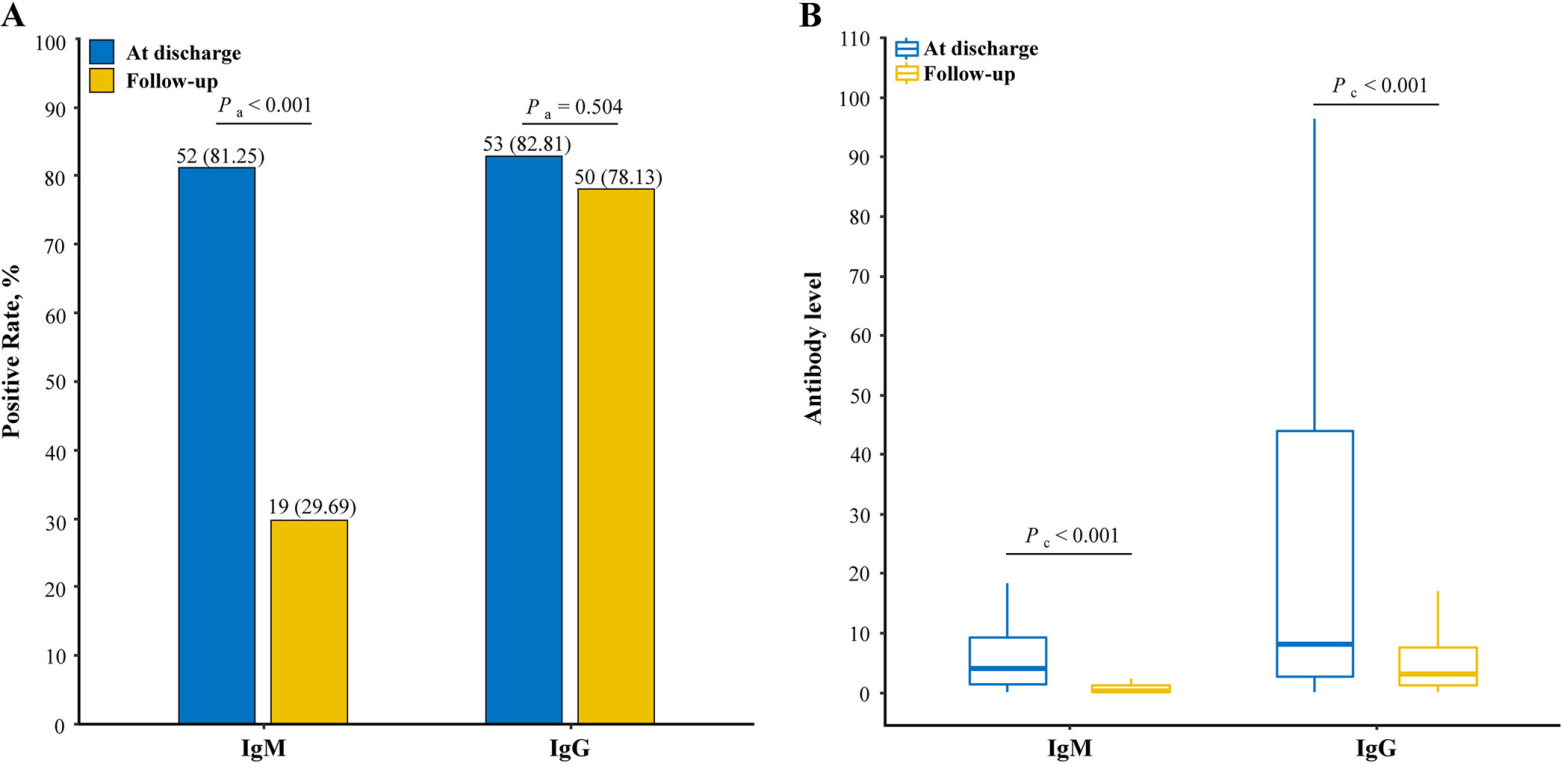
Seropositivity and antibody levels of IgM and IgG against SARS-CoV-2 at discharge and follow-up. (A) Positive rates of IgM and IgG presented in bar plots; 100% denotes 64 participants were IgM/IgG seropositive. (B) Chemiluminescent immunoassay measurements of IgM and IgG presented in box plots. *P*_a_ represents that the *P* value was produced by chi-square test, and *P*_c_ represents that the *P* value was produced by Wilcoxon rank sum (Mann-Whitney) test.

Of 284 participants, 64 were available for antibody testing when discharged from hospital. Compared with seropositivity and level of antibodies in the early phase, the decline tendency among these patients was reflected in this follow-up (Fig. 4). In the early phase, IgM was detectable in 52 (81.25%) patients and IgG in 53 (82.81%) patients. The level (S/CO) of IgM was 4.03 (IQR, 1.34 to 9.53) while IgG was 8.21 (IQR, 2.64 to 44.53) in 64 participants in the early phase. After at least 7 months, 29.69% with seropositive IgM and 78.13% with seropositive IgG were reported to be accompanied by the IgM level of 0.33 (IQR, 0.14 to 1.04) and the IgG level of 3.59 (IQR, 1.40 to 7.47). The significant decrease in seropositive rate and level could be observed in IgM (both *P *< 0.001). IgG-seropositive individuals remained at a high proportion (*P *= 0.504) but the level decreased from before (*P *< 0.001). The same findings were also observed in each type including 25 asymptomatic participants, 6 mild participants, and 33 normal participants (see Fig. S2 in the supplemental material).

### Neutralization against SARS-CoV-2 authentic viruses.

**FIG 5:**
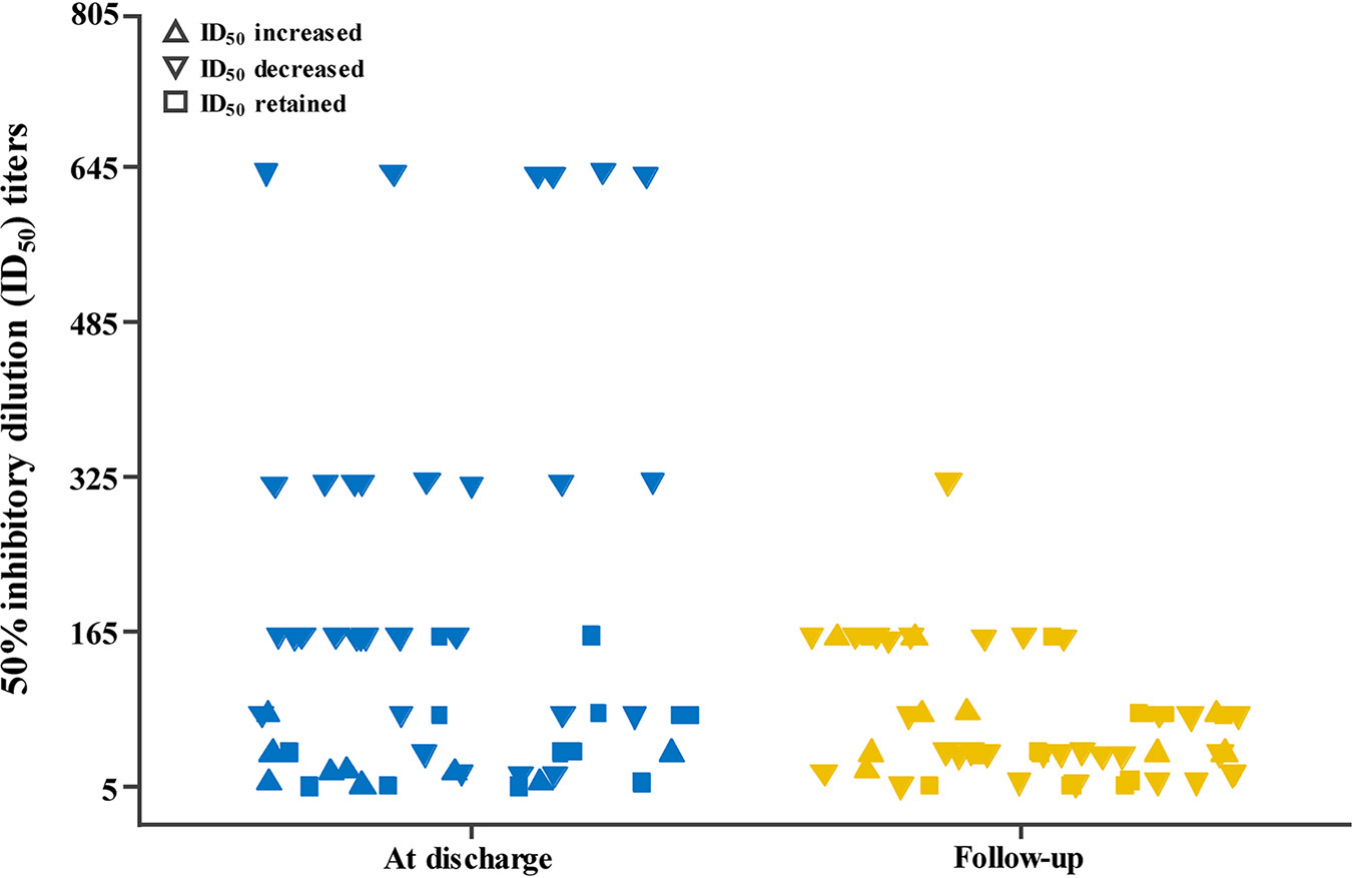
Neutralizing antibody (ID_50_) against SARS-CoV-2 authentic viruses at discharge and follow-up.

Plasma samples of 53 patients when discharged from hospital and in this follow-up were available for neutralizing antibody testing against WT viruses (Fig. 5; see also Table S1 in the supplemental material). The 50% inhibitory dilution (ID_50_) titers for samples when discharged from hospital (geometric mean titer [GMT], 82; 95% CI, 56 to 121) were significantly higher than those in this follow-up (geometric mean titer, 47; 95% CI: 35 to 63) (*P* < 0.001). Thirty-one of 53 plasma samples lost 3.7-fold neutralizing activity, 13 plasma samples retained activity, and 9 plasma samples increased 3.2-fold neutralizing activity over 199 days between sampling. No substantial differences in the extent of the change in neutralization activity were noted according to age, sex, or severity of disease (data not shown).

## DISCUSSION

In this study, we measured the levels of IgG/IgM and neutralizing antibodies against SARS-CoV-2 in reverse transcriptase PCR (RT-PCR)-confirmed COVID-19 patients 225.5 (IQR, 219 to 232) days after symptom onset, in order to verify persistent immunity against SARS-CoV-2 natural infection and identify the changing tendencies of antibodies in the long term. We found that compared to the level when participants discharge from hospital, the decline tendency did occur in both IgG and IgM over 7 months after symptom onset; however, the high seropositivity rate of IgG showed no significant difference. More importantly, the neutralizing activity of plasma samples decreased in this follow-up compared to those when discharged from hospital. These findings suggest that although SARS-CoV-2 natural infection is characterized by long-lived immune memory, the infection cannot build a strong protective barrier against reinfection.

As a result of the interaction between SARS-CoV-2 and the immune system in humans, the production of antibodies is so indispensable and impactful that it not only influences the outcome and prognosis of COVID-19 but also shapes the durative defense against extraneous invaders and helps develop the vaccination strategies to generate herd immunity (9). IgM reacting quickly in the early infected stage establishes a short-term response and IgG produced in the middle and later stages prolongs the immunity response, while neutralizing antibody is regarded as the gold standard for determining antibody efficacy. The long-lived immunity response of SARS-CoV-2 shown in our findings matched with that of severe acute respiratory syndrome-associated coronavirus (SARS-CoV), which binds the same receptor and shares approximately 79.6% genomic sequence identity with SARS-CoV-2 (10). Researchers have reported that IgG continuously existed for more than 2 years in SARS-recovered patients (11, 12). In addition, there are some previous researches that support our findings about the persistence of IgG. Iyer et al. recruited 343 patients and indicated that IgG persisted in patients through 90 days after symptom onset and IgM disappeared after a median time of 49 days (13). Isho et al. also said that anti-SARS-CoV-2 IgA and IgM stayed for a short time in serum and saliva, while IgG remained relatively stable up to 105 days from symptom onset (14). Also, we found that IgM in 25.70% of participants 6 to 9 months after symptom onset was still seropositive, which was significantly different from other pathogenic viruses, supplementing our understanding of IgM, whose seropositive rate decreased to around 55% in 9 to 10 weeks (15).

Interestingly, we found that the decline of antibodies was not sustained, and relatively stable phases called plateaus appeared between 162 and 282 days after symptom onset, which was similar to the plateaus of specific IgG against SARS 90 to 120 days after symptom onset in a longitudinal study of 176 SARS patients (12).

Notably, the antibody response patterns in the phases of acute infection and recovery were both associated with age and disease progression. There was evidence proving that in the acute phase, the risk of severe/critical illness was higher for elderly patients and the specific CD8^+^ T cell responses that were related to disease severity were damaged for the elderly patients (16–18). Unlike the immunological characteristics in the acute phase, we found that antibody response 162 to 282 days after symptom onset seemed to have no difference between the groups of 20- to 59-year-old patients and ≥60-year-old patients. On the contrary, children and adolescents are more likely to be spared COVID-19 or have mild symptoms, and their immune responses are not as violent as adults (16, 19–21). That was consistent with our finding of the lowest antibody level and positive rate in children and adolescents. Compared with the other three clinical types, severe/critical patients had higher antibody levels and positive rates, while the asymptomatic had the lowest levels and positive rates of IgG, consistent with previous studies (22, 23). The precise underlying cause-effect associations and relationships between IgM/IgG levels and severity of disease and age require further study to explore.

ID_50_ titers for samples when discharged from hospital (GMT, 82) decreased to a GMT of 47 at this follow-up. Many plasma samples decreased neutralizing activity, some plasma samples retained activity, and few plasma samples increased neutralizing activity between the two sampling periods. Anichini et al. found that neutralizing antibody titer after administration of a single dose of vaccine in previously infected patients was significantly higher than the titer after administration of a second dose of vaccine in previously uninfected patients (24). These findings provide evidence supporting that the administration of vaccine may be needed for those previously infected with SARS-CoV-2.

There were some limits in our study. First, the follow-up was only carried out at two time points, so we could not demonstrate the dynamic changes of antibodies after discharge, which may mislead the understanding of antibody linear decline. However, there have been many studies analyzing dynamic serologic responses in the acute phase (14, 15, 22, 25, 26). Second, limited by the number of participants of severe/critical type (*n* = 9), we could not explain further association between the antibody change trends and the severity of disease because of difficulty to measure the bias.

By observing the long-term antibody levels against SARS-CoV-2 and comparing antibody levels at two time points after symptom onset, we found that convalescent COVID-19 patients continued to present with high IgG seropositive rates; however, many plasma samples had decreased neutralizing activity. These findings stress the importance of vaccination and provide evidence to support the monitoring for neutralizing activity those previously infected with SARS-CoV-2.

In conclusion, for COVID-19 patients, age and disease severity not only affected the antibody response patterns in the phase of acute infection but also after recovery, and the elderly had different serological features in the two phases. The crucial facts were observed that, after 7 months, immune response of SARS-CoV-2-specific antibodies showed a promising performance; however, neutralizing antibodies decreased not to be able to maintain a protective barrier. These findings urge governments and departments of health to implement more active vaccination and imply that monitoring for neutralizing activity of SARS-CoV-2 in previously infected persons should be considered.

## MATERIALS AND METHODS

### Study design and participant enrollment.

Between 26 August 2020 and 28 October 2020 in Jiangsu province, China, a total of 284 cured patients were followed up who had been diagnosed with SARS-CoV-2 infection confirmed by real-time RT-PCR on nasal and/or pharyngeal swab specimens from 1 January to 20 March 2020 according to Diagnosis and Treatment Protocol for Novel Coronavirus Pneumonia released by the National Health Commission of the People’s Republic of China. These participants provided serum samples for antibody tests to check the presence and persistence of immunity to SARS-CoV-2 and accepted chest CT to figure out the damage influence on lungs 7 months or longer after symptom onset. Additionally, we reviewed documents on epidemiological investigation and medical records when the participants were in hospital for treatment from the China Information System for Disease Control and Prevention (CISDC). Of the 284 patients, 64 participants were tested for antibodies when discharged from hospital.

### Detection of IgG and IgM against SARS-CoV-2.

Serum samples were collected from the participants to measure the levels of IgG and IgM against SARS-CoV-2 using the following commercial kits: Novel Coronavirus (2019-nCoV) IgM/G antibody diagnostic kit (plate CLIA) supplied by Bioscience Co. (China National Medical Products Administration, approval numbers 20203400183 [IgG] and 20203400182 [IgM]) on an automated magnetic chemiluminescence analyzer (Axceed 260; Bioscience). According to the manufacturer’s instructions, based on a double-antibody sandwich immunoassay, the detection antibody is an alkaline phosphatase-conjugated anti-human IgG/IgM antibody, and the recombinant antigens contain the nucleoprotein and a peptide from the spike protein of SARS-CoV-2, which are conjugated with fluorescein isothiocyanate (FITC) and immobilized on anti-FITC antibody-conjugated magnetic particles. Associated positively with the measured chemiluminescence values, the IgM/IgG titers are presented as chemiluminescence values divided by the cutoff (S/CO). If the S/CO value is >1, the sample is considered seropositive for IgM or IgG, while if the S/CO value is ≤1, the sample is considered seronegative for IgM or IgG. The detection performance of the commercial magnetic chemiluminescence enzyme immunoassay (MCLIA) kit has been reported by the manufacturer as follows: the sensitivity and specificity for IgG are 87.23% (95% CI, 82.77 to 90.90%) and 99.25% (95% CI, 97.83 to 99.85%) and for IgM are 88.30% (95% CI, 83.96 to 91.81%) and 99.50% (95% CI, 98.21 to 99.94%).

### Authentic SARS-CoV-2 microplate neutralization.

The experiments were performed in a biosafety level 3 (BSL3) laboratory in Jiangsu Provincial Center for Diseases Control and Prevention, Jiangsu, China, as described previously (27). The frozen SARS-CoV-2 strain (SARS-CoV-2/human/CHN/Changzhou_JS27/2020; GenBank accession no. MT534630) was thawed in a 37°C water bath and propagated for one passage using Vero-E6 cells. Virus infectious titer was determined by the 50% tissue culture infective dose (TCID_50_) based on microscopic observation of cytopathic effect (CPE) assay on Vero-E6 cells. An endpoint dilution microplate neutralization assay was performed to measure the neutralization activity of convalescent plasma samples. Plasma serum samples were heat-inactivated for 30 min at 56°C and subjected to successive 2-fold dilution starting from 1:10 to 1:5,120. Triplicates of each dilution were incubated with SARS-CoV-2 in Dulbecco modified Eagle medium (DMEM) with 2% inactivated fetal calf serum (FCS) for 1 h at 37°C. Postincubation, the virus-antibody mixture was transferred onto a monolayer of Vero-E6 cells grown overnight. The cells were incubated with the mixture for 3 days. CPE of viral infection was visually scored under an inverted microscope for each well in a blinded fashion by two independent observers. The neutralizing titer is the highest sample dilution that protects at least 50% of Vero-E6 cells from CPE. If no neutralization reaction was observed at the initial serum dilution (1:10), an arbitrary titer of 5 was reported.

### Data source.

The clinical types of participants were decided on by physicians upon admission on basis of the symptoms and severity of disease according to the Diagnosis and Treatment Protocol for Novel Coronavirus Pneumonia (28). COVID-19 patients without any relevant clinical symptoms in the preceding 14 days and during hospitalization, with mild clinical symptoms but without pneumonia manifestation found by imaging, and with typical clinical symptoms (fever and respiratory tract symptoms, etc.) and pneumonia manifestation found by imaging were classified as asymptomatic, mild, and normal cases, respectively. Severe/critical COVID-19 patients met any of the following criteria: (i) respiratory distress, respiratory rates of ≥30 breaths/min; (ii) percutaneous oxygen saturation (SpO_2_) of ≤93% at rest; (iii) arterial oxygen tension/inspiratory oxygen fraction ratio (PaO_2_/FiO_2_) of ≤300 mm Hg; (iv) greater than 50% lesion progression within 24 to 48 h in pulmonary imaging; (v) respiratory failure requiring mechanical ventilation; (vi) shock; and (vii) complications from other organ failure requiring monitoring and treatment in the intensive care unit (ICU). During hospitalization, demographic characteristics, medical interventions, and complications were documented.

### Statistical analysis.

Continuous variables were expressed as means ± standard deviation (SD) and tested using one-way analysis of variance (ANOVA) or the Wilcoxon rank sum (Mann-Whitney) test as appropriate; categorical variables were presented in percentages and tested using the chi-square test and Fisher's exact test as appropriate. The medians and quartiles of chemiluminescence values for anti-SARS-CoV-2 antibodies were represented in box plots, and the prevalence and positive/total numbers were shown in lines and bar plots. The box plot shows medians represented as middle lines and third and first quartiles are represented by the box, while the whiskers above and below the box show the difference values between 1.5× the interquartile range (IQR) and third and first quartiles. *P* values for the comparison of the reciprocal neutralization titers at 50% inhibitory dilution (ID_50_) are calculated with the use of the Wilcoxon signed-rank test. All statistical analyses were conducted with the R software (version 4.0.3.). Differences were considered to be statistically significant when the *P* value was 0.05 or less. All statistical tests were two-sided.

The study was approved by the Institutional Review Board of Nanjing Medical University. All participants have provided written informed consent for demographic characteristics, physical examinations, medical records, and blood sample tests.

## References

[1] Wiersinga WJ, Rhodes A, Cheng AC, Peacock SJ, Prescott HC. 2020. Pathophysiology, transmission, diagnosis, and treatment of coronavirus disease 2019 (COVID-19): a review. JAMA 324:782–793. doi:10.1001/jama.2020.12839. 32648899

[2] Jeyanathan M, Afkhami S, Smaill F, Miller MS, Lichty BD, Xing Z. 2020. Immunological considerations for COVID-19 vaccine strategies. Nat Rev Immunol 20:615–632. doi:10.1038/s41577-020-00434-6. 32887954 PMC7472682

[3] World Health Organization. 2021. Weekly operational update on COVID-19 - 26 January 2021. World Health Organization, Geneva, Switzerland. https://www.who.int/publications/m/item/weekly-operational-update-on-covid-19---26-january-2021.

[4] Fontanet A, Cauchemez S. 2020. COVID-19 herd immunity: where are we? Nat Rev Immunol 20:583–584. doi:10.1038/s41577-020-00451-5. 32908300 PMC7480627

[5] Krammer F. 2020. SARS-CoV-2 vaccines in development. Nature 586:516–527. doi:10.1038/s41586-020-2798-3. 32967006

[6] Galipeau Y, Greig M, Liu G, Driedger M, Langlois MA. 2020. Humoral responses and serological assays in SARS-CoV-2 infections. Front Immunol 11:610688. doi:10.3389/fimmu.2020.610688. 33391281 PMC7775512

[7] Rogers TF, Zhao F, Huang D, Beutler N, Burns A, He WT, Limbo O, Smith C, Song G, Woehl J, Yang L, Abbott RK, Callaghan S, Garcia E, Hurtado J, Parren M, Peng L, Ramirez S, Ricketts J, Ricciardi MJ, Rawlings SA, Wu NC, Yuan M, Smith DM, Nemazee D, Teijaro JR, Voss JE, Wilson IA, Andrabi R, Briney B, Landais E, Sok D, Jardine JG, Burton DR. 2020. Isolation of potent SARS-CoV-2 neutralizing antibodies and protection from disease in a small animal model. Science 369:956–963. doi:10.1126/science.abc7520. 32540903 PMC7299280

[8] Huang C, Huang L, Wang Y, Li X, Ren L, Gu X, Kang L, Guo L, Liu M, Zhou X, Luo J, Huang Z, Tu S, Zhao Y, Chen L, Xu D, Li Y, Li C, Peng L, Li Y, Xie W, Cui D, Shang L, Fan G, Xu J, Wang G, Wang Y, Zhong J, Wang C, Wang J, Zhang D, Cao B. 2021. 6-month consequences of COVID-19 in patients discharged from hospital: a cohort study. Lancet 397:220–232. doi:10.1016/S0140-6736(20)32656-8. 33428867 PMC7833295

[9] Velikova TV, Kotsev SV, Georgiev DS, Batselova HM. 2020. Immunological aspects of COVID-19: What do we know? World J Biol Chem 11:14–29. doi:10.4331/wjbc.v11.i2.14. 33024515 PMC7520644

[10] Jiang S, Hillyer C, Du L. 2020. Neutralizing antibodies against SARS-CoV-2 and other human coronaviruses. Trends Immunol 41:355–359. doi:10.1016/j.it.2020.03.007. 32249063 PMC7129017

[11] Cao WC, Liu W, Zhang PH, Zhang F, Richardus JH. 2007. Disappearance of antibodies to SARS-associated coronavirus after recovery. N Engl J Med 357:1162–1163. doi:10.1056/NEJMc070348. 17855683

[12] Wu LP, Wang NC, Chang YH, Tian XY, Na DY, Zhang LY, Zheng L, Lan T, Wang LF, Liang GD. 2007. Duration of antibody responses after severe acute respiratory syndrome. Emerg Infect Dis 13:1562–1564. doi:10.3201/eid1310.070576. 18258008 PMC2851497

[13] Iyer AS, Jones FK, Nodoushani A, Kelly M, Becker M, Slater D, Mills R, Teng E, Kamruzzaman M, Garcia-Beltran WF, Astudillo M, Yang D, Miller TE, Oliver E, Fischinger S, Atyeo C, Iafrate AJ, Calderwood SB, Lauer SA, Yu J, Li Z, Feldman J, Hauser BM, Caradonna TM, Branda JA, Turbett SE, LaRocque RC, Mellon G, Barouch DH, Schmidt AG, Azman AS, Alter G, Ryan ET, Harris JB, Charles RC. 2020. Persistence and decay of human antibody responses to the receptor binding domain of SARS-CoV-2 spike protein in COVID-19 patients. Sci Immunol 5:eabe0367. doi:10.1126/sciimmunol.abe0367. 33033172 PMC7857394

[14] Isho B, Abe KT, Zuo M, Jamal AJ, Rathod B, Wang JH, Li Z, Chao G, Rojas OL, Bang YM, Pu A, Christie-Holmes N, Gervais C, Ceccarelli D, Samavarchi-Tehrani P, Guvenc F, Budylowski P, Li A, Paterson A, Yue FY, Marin LM, Caldwell L, Wrana JL, Colwill K, Sicheri F, Mubareka S, Gray-Owen SD, Drews SJ, Siqueira WL, Barrios-Rodiles M, Ostrowski M, Rini JM, Durocher Y, McGeer AJ, Gommerman JL, Gingras AC. 2020. Persistence of serum and saliva antibody responses to SARS-CoV-2 spike antigens in patients with COVID-19. Sci Immunol 5:eabe5511. doi:10.1126/sciimmunol.abe5511. 33033173 PMC8050884

[15] Fu Y, Li Y, Guo E, He L, Liu J, Yang B, Li F, Wang Z, Li Y, Xiao R, Liu C, Huang Y, Wu X, Lu F, You L, Qin T, Wang C, Li K, Wu P, Ma D, Sun C, Chen G. 2021. Dynamics and correlation among viral positivity, seroconversion, and disease severity in COVID-19: a retrospective study. Ann Intern Med 174:453–461. doi:10.7326/M20-3337. 33284684 PMC7745119

[16] Docherty AB, Harrison EM, Green CA, Hardwick HE, Pius R, Norman L, Holden KA, Read JM, Dondelinger F, Carson G, Merson L, Lee J, Plotkin D, Sigfrid L, Halpin S, Jackson C, Gamble C, Horby PW, Nguyen-Van-Tam JS, Ho A, Russell CD, Dunning J, Openshaw PJ, Baillie JK, Semple MG, ISARIC4C investigators. 2020. Features of 20 133 UK patients in hospital with covid-19 using the ISARIC WHO clinical characterisation protocol: prospective observational cohort study. BMJ 369:m1985. doi:10.1136/bmj.m1985. 32444460 PMC7243036

[17] Rydyznski Moderbacher C, Ramirez SI, Dan JM, Grifoni A, Hastie KM, Weiskopf D, Belanger S, Abbott RK, Kim C, Choi J, Kato Y, Crotty EG, Kim C, Rawlings SA, Mateus J, Tse LPV, Frazier A, Baric R, Peters B, Greenbaum J, Ollmann Saphire E, Smith DM, Sette A, Crotty S. 2020. Antigen-specific adaptive immunity to SARS-CoV-2 in acute COVID-19 and associations with age and disease severity. Cell 183:996–1012. doi:10.1016/j.cell.2020.09.038. 33010815 PMC7494270

[18] Yang X, Yu Y, Xu J, Shu H, Xia J, Liu H, Wu Y, Zhang L, Yu Z, Fang M, Yu T, Wang Y, Pan S, Zou X, Yuan S, Shang Y. 2020. Clinical course and outcomes of critically ill patients with SARS-CoV-2 pneumonia in Wuhan, China: a single-centered, retrospective, observational study. Lancet Respir Med 8:475–481. doi:10.1016/S2213-2600(20)30079-5. 32105632 PMC7102538

[19] Lu X, Zhang L, Du H, Zhang J, Li YY, Qu J, Zhang W, Wang Y, Bao S, Li Y, Wu C, Liu H, Liu D, Shao J, Peng X, Yang Y, Liu Z, Xiang Y, Zhang F, Silva RM, Pinkerton KE, Shen K, Xiao H, Xu S, Wong GWK, Chinese Pediatric Novel Coronavirus Study Team. 2020. SARS-CoV-2 infection in children. N Engl J Med 382:1663–1665. doi:10.1056/NEJMc2005073. 32187458 PMC7121177

[20] Pierce CA, Preston-Hurlburt P, Dai Y, Aschner CB, Cheshenko N, Galen B, Garforth SJ, Herrera NG, Jangra RK, Morano NC, Orner E, Sy S, Chandran K, Dziura J, Almo SC, Ring A, Keller MJ, Herold KC, Herold BC. 2020. Immune responses to SARS-CoV-2 infection in hospitalized pediatric and adult patients. Sci Transl Med 12:eabd5487. doi:10.1126/scitranslmed.abd5487. 32958614 PMC7658796

[21] Weisberg SP, Connors TJ, Zhu Y, Baldwin MR, Lin WH, Wontakal S, Szabo PA, Wells SB, Dogra P, Gray J, Idzikowski E, Stelitano D, Bovier FT, Davis-Porada J, Matsumoto R, Poon MML, Chait M, Mathieu C, Horvat B, Decimo D, Hudson KE, Zotti FD, Bitan ZC, La Carpia F, Ferrara SA, Mace E, Milner J, Moscona A, Hod E, Porotto M, Farber DL. 2021. Distinct antibody responses to SARS-CoV-2 in children and adults across the COVID-19 clinical spectrum. Nat Immunol 22:25–31. doi:10.1038/s41590-020-00826-9. 33154590 PMC8136619

[22] Li K, Huang B, Wu M, Zhong A, Li L, Cai Y, Wang Z, Wu L, Zhu M, Li J, Wang Z, Wu W, Li W, Bosco B, Gan Z, Qiao Q, Wu J, Wang Q, Wang S, Xia X. 2020. Dynamic changes in anti-SARS-CoV-2 antibodies during SARS-CoV-2 infection and recovery from COVID-19. Nat Commun 11:6044. doi:10.1038/s41467-020-19943-y. 33247152 PMC7699636

[23] Long QX, Tang XJ, Shi QL, Li Q, Deng HJ, Yuan J, Hu JL, Xu W, Zhang Y, Lv FJ, Su K, Zhang F, Gong J, Wu B, Liu XM, Li JJ, Qiu JF, Chen J, Huang AL. 2020. Clinical and immunological assessment of asymptomatic SARS-CoV-2 infections. Nat Med 26:1200–1204. doi:10.1038/s41591-020-0965-6. 32555424

[24] Anichini G, Terrosi C, Gandolfo C, Gori Savellini G, Fabrizi S, Miceli GB, Cusi MG. 2021. SARS-CoV-2 antibody response in persons with past natural infection. N Engl J Med 385:90–92. doi:10.1056/NEJMc2103825. 33852796 PMC8063888

[25] Hartley GE, Edwards ESJ, Aui PM, Varese N, Stojanovic S, McMahon J, Peleg AY, Boo I, Drummer HE, Hogarth PM, O'Hehir RE, van Zelm MC. 2020. Rapid generation of durable B cell memory to SARS-CoV-2 spike and nucleocapsid proteins in COVID-19 and convalescence. Sci Immunol 5:eabf8891. doi:10.1126/sciimmunol.abf8891. 33443036 PMC7877496

[26] Lagunas-Rangel FA, Chavez-Valencia V. 2021. What do we know about the antibody responses to SARS-CoV-2? Immunobiology 226:152054. doi:10.1016/j.imbio.2021.152054. 33524881 PMC7826124

[27] Li J, Hui A, Zhang X, Yang Y, Tang R, Ye H, Ji R, Lin M, Zhu Z, Türeci Ö, Lagkadinou E, Jia S, Pan H, Peng F, Ma Z, Wu Z, Guo X, Shi Y, Muik A, Şahin U, Zhu L, Zhu F. 2021. Safety and immunogenicity of the SARS-CoV-2 BNT162b1 mRNA vaccine in younger and older Chinese adults: a randomized, placebo-controlled, double-blind phase 1 study. Nat Med 27:1062–1070. doi:10.1038/s41591-021-01330-9. 33888900

[28] National Health Commission of the People’s Republic of China. 2020. Diagnosis and treatment protocol for COVID-19 (trial version 6). National Health Commission of the People’s Republic of China, Beijing, China. http://www.nhc.gov.cn/yzygj/s7653p/202002/8334a8326dd94d329df351d7da8aefc2.shtml.

