# Supplementary material for: Kinetics of SARS-CoV-2 Specific and Neutralizing Antibodies over Seven Months after Symptom Onset in COVID-19 Patients
Source: Microbiol Spectr. 2021 Sep 22;9(2):e00590-21. doi: 10.1128/Spectrum.00590-21 (PMC8557935; doi:10.1128/Spectrum.00590-21)

Supplemental Table 1. The exact values of ID_{50} titers of 53 COVID-19 convalescent patients at discharge and seven months after symptom onset.

Code	ID_{50} at discharge	ID_{50} at Follow-up	Change	Gender	Age
1	10	20	Increased	Female	53
2	20	40	Increased	Female	53
3	20	10	Decreased	Female	62
6	160	160	Retained	Male	61
7	320	160	Decreased	Female	51
8	640	160	Decreased	Female	45
9	80	160	Increased	Female	42
10	80	80	Retained	Female	78
11	80	10	Decreased	Male	30
12	80	80	Retained	Male	50
13	160	40	Decreased	Female	63
14	40	40	Retained	Female	57
16	20	5	Decreased	Female	65
17	640	160	Decreased	Female	57
18	160	80	Decreased	Female	53
19	160	160	Retained	Female	63
21	20	10	Decreased	Male	56
22	160	40	Decreased	Male	59
23	320	160	Decreased	Female	59
24	640	160	Decreased	Male	58
25	640	80	Decreased	Male	58
29	40	40	Retained	Male	40
30	40	10	Decreased	Female	40
31	320	160	Decreased	Male	59
32	640	320	Decreased	Male	54
33	640	160	Decreased	Male	77
34	80	80	Retained	Female	55
35	160	40	Decreased	Male	29
36	320	160	Decreased	Female	51
37	320	40	Decreased	Female	48

38	320	40	Decreased	Female	49
39	160	40	Decreased	Female	33
40	80	40	Decreased	Male	63
41	10	10	Retained	Female	14
42	5	5	Retained	Male	31
43	80	40	Decreased	Male	33
44	320	80	Decreased	Female	48
45	40	40	Retained	Female	21
47	40	80	Increased	Female	48
49	160	20	Decreased	Female	29
50	160	20	Decreased	Female	23
51	320	80	Decreased	Female	26
52	160	40	Decreased	Female	45
53	5	5	Retained	Male	32
54	80	20	Decreased	Female	39
55	80	80	Retained	Female	73
56	5	40	Increased	Male	79
57	160	40	Decreased	Female	63
59	20	80	Increased	Male	57
60	40	80	Increased	Male	27
61	20	160	Increased	Male	67
62	5	5	Retained	Female	24
63	10	40	Increased	Male	51

Supplemental Figure 1. Number and proportion of convalescent individuals with different disease severity in each quartile of days after symptom onset. The numbers of four disease severity kinds of all 284 (A), IgM positive (B) and IgG positive (C) convalescent participants are shown in the different color bar while percentage values denote the corresponding proportion.

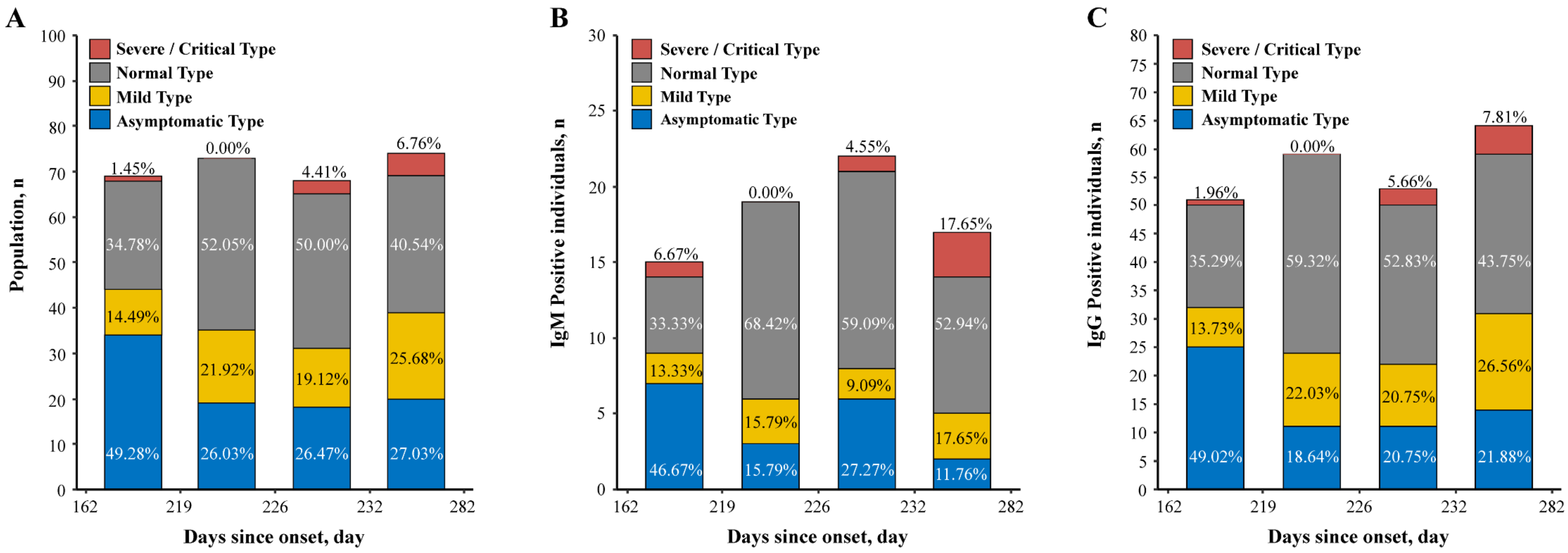

Supplemental Figure 2. Seropositivity and antibody chemiluminescence values of IgM and IgG of different disease severity individuals against SARS-CoV-2 at discharge and follow-up. (A) and (B): positive rates of IgM and IgG in different disease severity types presented in bar plots respectively; (C) and (D): chemiluminescent immunoassay measurements of IgM and IgG in different disease severity types presented in box plots respectively. In 64 convalescent individuals tested at discharge and follow-up, no one belonged to severe/critical type. P_a represents that the P value was produced by Chi-square test; p_b represents that the P value was produced by Fisher's exact test; p_c represents that the P value was produced by Wilcoxon rank-sum (Mann-Whitney) test.

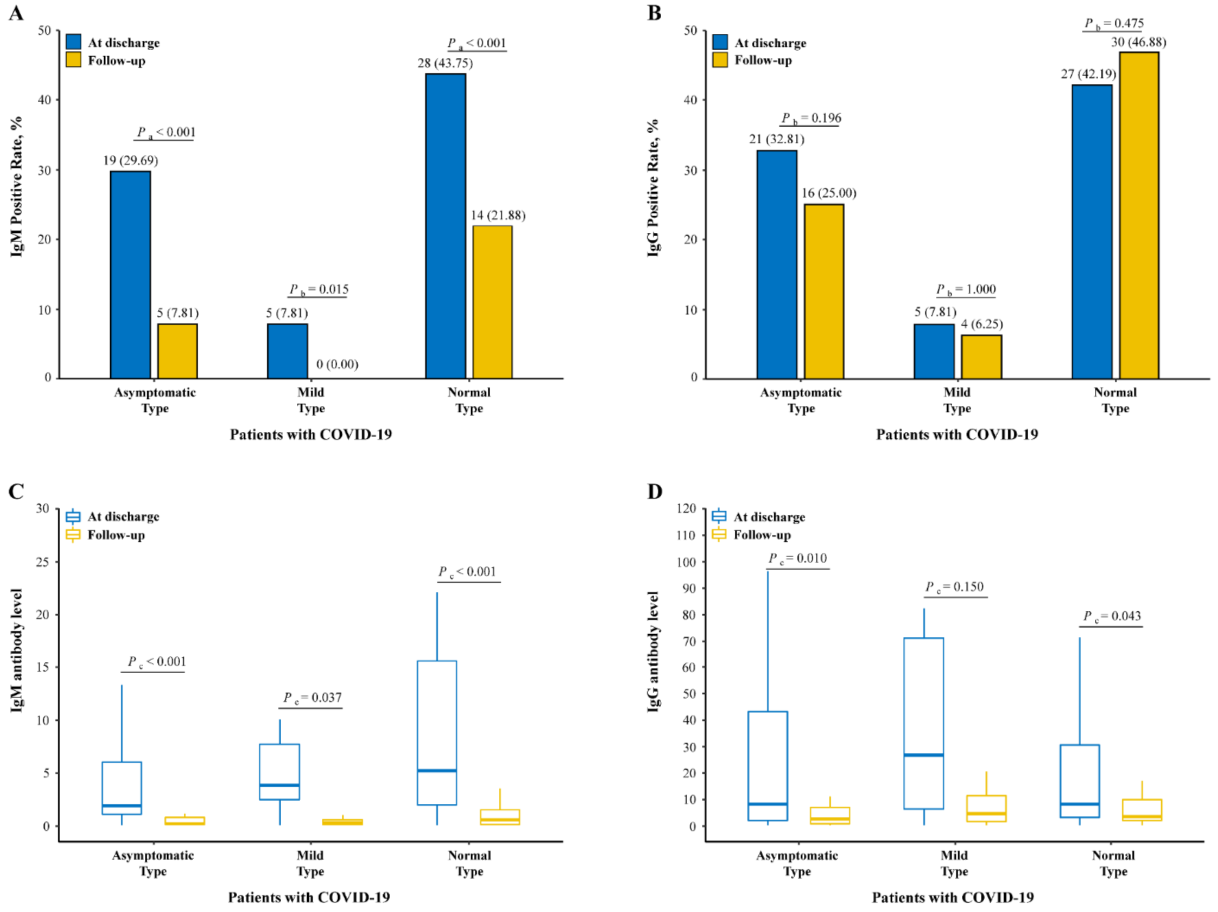

Supplement: SUPPLEMENTAL FILE 1 — Supplemental material. Download SPECTRUM00590-21_Supp_1_seq10.pdf, PDF file, 0.3 MB [file spectrum00590-21_supp_1_seq10.pdf]
